# Investigation of the Saturation Pulse Artifact in Non-Enhanced MR Angiography of the Lower Extremity Arteries at 7 Tesla

**DOI:** 10.1371/journal.pone.0119845

**Published:** 2015-03-18

**Authors:** Sören Johst, Stefan Maderwald, Anja Fischer, Harald H. Quick, Mark E. Ladd, Stephan Orzada

**Affiliations:** 1 Erwin L. Hahn Institute for Magnetic Resonance Imaging, University Duisburg-Essen, Essen, Germany; 2 Department of Diagnostic and Interventional Radiology and Neuroradiology, University Hospital Essen, University Duisburg-Essen, Essen, Germany; 3 High Field and Hybrid MR Imaging, University Hospital Essen, University Duisburg-Essen, Essen, Germany; 4 Medical Physics in Radiology, German Cancer Research Center (dkfz), Heidelberg, Germany; University of Washington School of Medicine, UNITED STATES

## Abstract

When performing non-enhanced time-of-flight MR angiography of the lower extremity arteries at 7 T with cardiac triggering, the acquisition time is a crucial consideration. Therefore, in previous studies, saturation RF pulses were applied only every second TR. In the axial source images a slight artifact with an appearance similar to aliasing could be observed. The purpose of this study was to investigate the origin of this artifact. The reason for the artifact is supposed to be related to the two effective TRs during acquisition caused by the sparsely applied saturation RF pulse. Several sequence variants were simulated and implemented within the sequence source code to examine this hypothesis. An adaptation of the excitation flip angles for each TR as well as a correction factor for the k-space data was calculated. Additionally, a different ordering of the k-space data during acquisition was implemented as well as the combination of the latter with the k-space correction factor. The observations from the simulations were verified using both a static and a flow phantom and, finally, in a healthy volunteer using the same measurement setup as in previous volunteer and patient studies. Of all implemented techniques, only the reordering of the k-space was capable of suppressing the artifact almost completely at the cost of creating a ringing artifact. The phantom measurements showed the same results as the simulations and could thus confirm the hypothesis regarding the origin of the artifact. This was additionally verified in the healthy volunteer. The origin of the artifact could be confirmed to be the periodic signal variation caused by two effective TRs during acquisition.

## Introduction

In recent studies, non-contrast-enhanced (ne) MR angiography (MRA) sequences have been evaluated as an alternative to digital subtraction angiography (DSA) and contrast-enhanced MRA for the diagnosis of lower extremity vascular diseases [1–6]. Using ne-MRA techniques has the clear advantage of being non-invasive and avoiding application of potentially harmful ionizing radiation. Furthermore, cases of Nephrogenic Systemic Fibrosis (NSF) have been reported after the administration of gadolinium(Gd)-based contrast agents [7, 8]. Initial work at an increased magnetic field strength of 7 T indicates further potential for the usage of non-contrast-enhanced time-of-flight (TOF) MRA sequences, as the visibility of the vasculature increases [9, 10], and the feasibility of ne-MRA of the lower extremity arteries at 7 T was recently demonstrated in healthy volunteers and patients [11–13].

**Fig 1:**
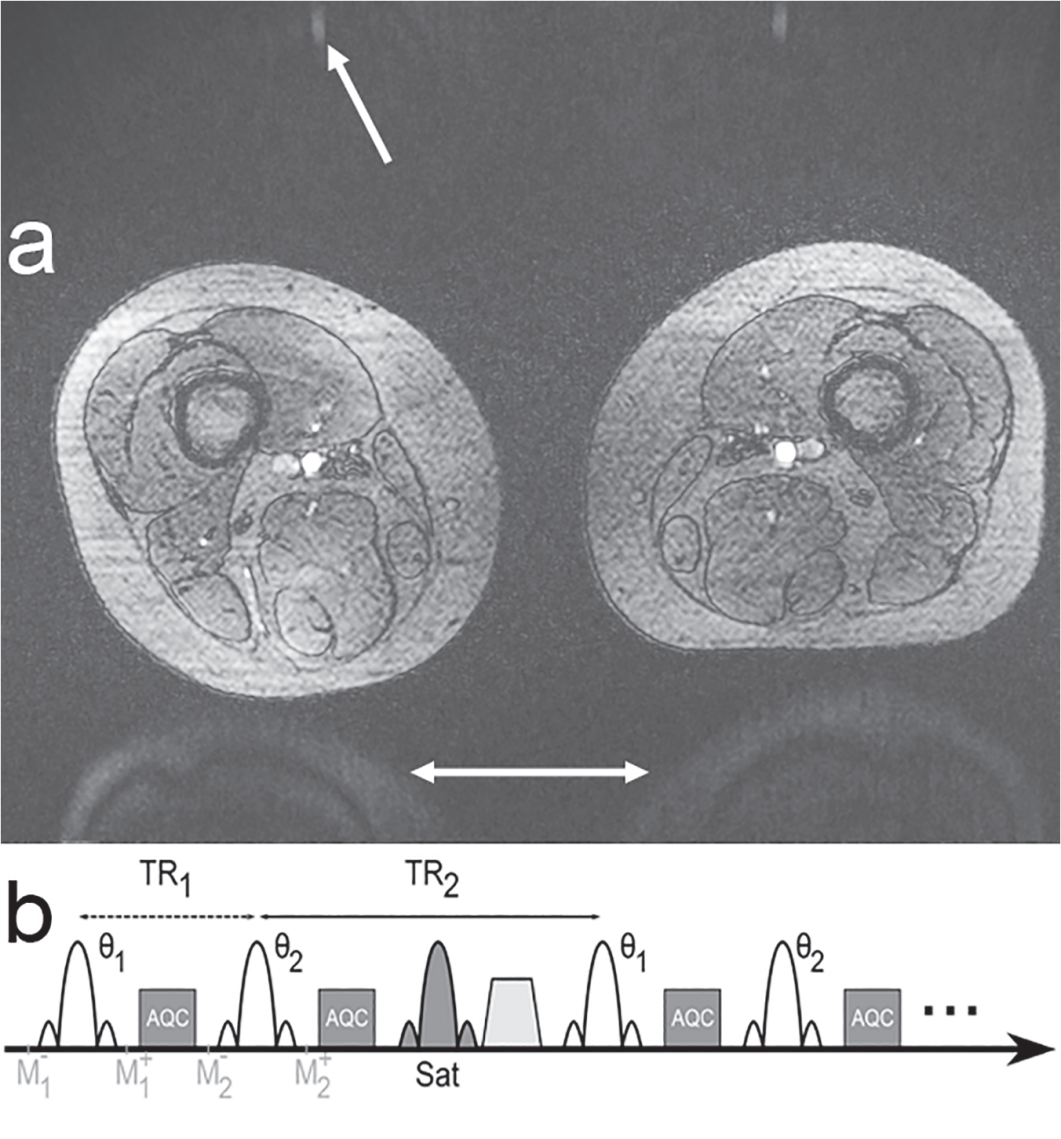
Artifact and sequence diagram. Axial source image of MRA of the lower extremities at the level of the upper thighs (a). Imaging with two effective TRs leads to an artifact that appears similar to aliasing (arrows). A diagram of the TFL sequence implementation shows the variation in effective TR due to the saturation pulse block preceding every second excitation (b).

In a recently published work, a modified turbo-FLASH (TFL) sequence was utilized for non-contrast-enhanced imaging of the lower extremity arteries [12] (Fig. 1A). To reduce known B_1_ inhomogeneities at 7 T [14], Time-Interleaved Acquisition of Modes (TIAMO) [15, 16] was integrated into the sequence. The principle of TIAMO is to excite at least two different B_1_ transmission modes using static radiofrequency (RF) shimming in an interleaved acquisition. In so doing, overall signal homogeneity can be improved by exploiting the complementary radiofrequency patterns of the different transmission modes. Data from e.g. two acquisitions are not just averaged, but reconstructed the same way as if twice the number of receive coils would have been used, additionally allowing higher acceleration factors to be used to reduce the scan time increase arising from multiple acquisitions. Finally, considerably more homogeneous images can be obtained. To avoid periodic variation in the signal intensity of the arteries that was observed in [11], acoustic cardiac triggering via a phonocardiogram was additionally used to synchronize the sequence with the heartbeat [17–19].

As mainly the heart rate defines the total acquisition time, one complete slice (one TR) should preferably be acquired between two heartbeats to maintain a reasonable acquisition time. TRs shorter than the time interval between two heartbeats lead to significantly shorter total measurement times. Thus, the shorter the TR, the higher the heart rate that can be acquired without measurement time prolongation. One way to decrease TR is to apply RF pulses for the suppression of signal from the venous system only sparsely [20, 21]. Volunteer measurements preceding the examinations in [11–13] showed that RF pulses for venous suppression need to be applied at least every second TR (Fig. 1B) to suppress the veins sufficiently using the chosen acquisition parameters. Measurement times with this method for the acquisition of one station with 60 slices amounted on average to 2 min 54, depending on the heart rate of the volunteer / patient (1 min 24 s without triggering) [12]. The application of the saturation RF pulse together with its spoiling gradient every other TR leads to two different effective TRs. This led to an unexpected artifact in axial slices that appears similar to aliasing (Fig. 1A). In the example shown, artifact intensity was approximately 50% up to 67% of the background tissue signal and approximately 20% of the arterial signal.

This publication examines the origin and nature of the observed aliasing-like artifact. The artifact is hypothesized to be caused by the periodic signal variation induced by the two alternating different effective TRs: every second line is acquired with slightly weaker signal, which leads to an attenuated aliasing artifact—similar to the aliasing which would appear if these lines would not be acquired at all. Therefore, different sequence variants were simulated to examine their impact on the artifact. In the next step the variants were implemented within the sequence source code and compared in measurements with a static setup, a flow phantom, and finally in-vivo. Understanding the nature of the artifact is important for future modifications and further developments of the used sequence variant.

## Materials and Methods

### Ethics Statement

The study was conducted in conformance with the Declaration of Helsinki and approved by the Ethics Commission of the Medical Faculty of the University Duisburg-Essen (study number 11-4898-BO). Written informed consent was obtained from each volunteer before the examination.

The use of different TRs during acquisition can be found e.g. in balanced steady-state free precession (SSFP) sequences [22, 23] or in the actual flip angle imaging (AFI) method [24, 25]. Just as in the modified TFL sequence presented here, in the original version of the AFI sequence [24] two alternating TRs generate two different signals that are used to calculate a flip angle map. Compared to the AFI sequence, the present TFL sequence acquires only half the lines, as in AFI every line is collected twice and two complete images are reconstructed from the two signals individually. This means that the signal formulas given in the publication by Yarnykh [24] can be used to simulate the observed artifact.

### Simulations

**Fig 2:**
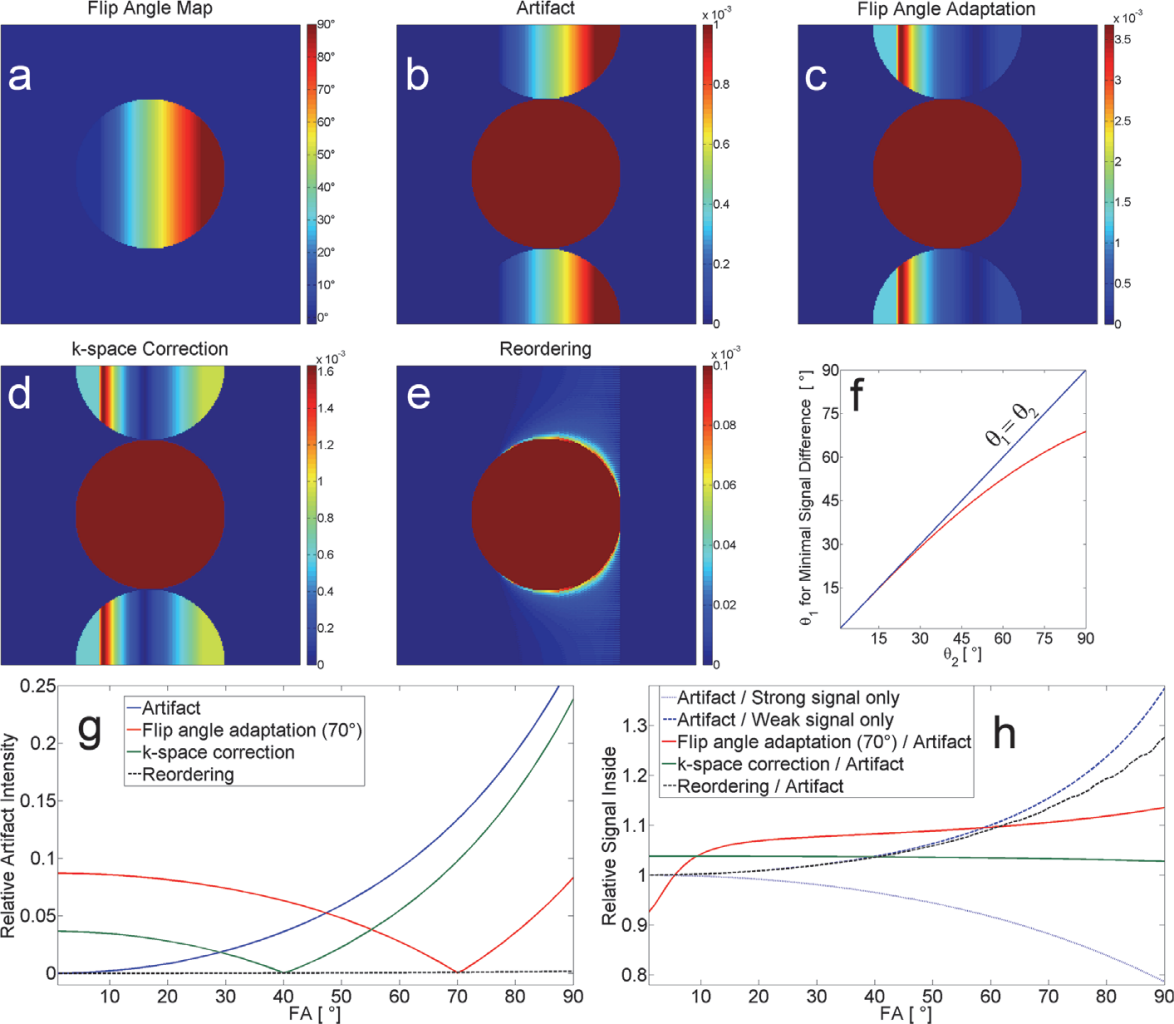
Simulation results. Simulations were performed with flip angle map (a) to illustrate the artifact (b). (c)-(e) show different sequence variants: flip angle adaptation (c) with the flip angle calculated for minimal signal difference (red line in f), correction factor applied in k-space (d), and low-pass filter-like reordering of k-space (e). The combination of reordering and k-space correction factor was nearly identical to the reordering alone and is thus not shown here. (g) shows artifact signal intensity relative to corresponding signal inside the phantom. (h) shows signal intensity within the phantom relative to the image with artifact (red, green, and dotted black lines) as well as the intensity of the artifact image relative to an image created with strong / weak signal only (dotted and dashed blue lines). Scaling of the images (b-e) was oriented on the corresponding maximum artifact signal. Hence, the signal inside the phantom is cut off and appears homogeneous.

To simulate the artifact, a flip-angle map inside a circular phantom including a linear gradient from 1° to 90° within and 0° outside (Fig. 2A) was defined in Matlab (R2010a, Mathworks, Natick, USA). This map was used to calculate two images based on the alternating signals produced by excitation with two alternating TRs [24]:

$$S_{1}=M_{0}\;\cdot \frac{1+e^{-\frac{T_{R,2}}{T_{1}}}\cdot \left(\left(1-e^{-\frac{T_{R,1}}{T_{1}}}\right)\cdot \text{cos}\;\theta -1\right)}{1-{\text{cos}}^{2}\;\theta \cdot e^{-\frac{T_{R,1}+T_{R,2}}{T_{1}}}}\;\cdot \;e^{-\frac{T_{E}}{T_{2}^{*}}}\;\cdot \;\text{sin}\;\theta$$ (Eq. 1)

and

$$S_{2}=M_{0}\;\cdot \frac{1+e^{-\frac{T_{R,1}}{T_{1}}}\cdot \left(\left(1-e^{-\frac{T_{R,2}}{T_{1}}}\right)\cdot \text{cos}\;\theta -1\right)}{1-{\text{cos}}^{2}\;\theta \cdot e^{-\frac{T_{R,1}+T_{R,2}}{T_{1}}}}\;\cdot \;e^{-\frac{T_{E}}{T_{2}^{*}}}\;\cdot \;\text{sin}\;\theta$$ (Eq. 2)

Both images were Fourier transformed (FT) and a new k-space was formed by taking every even line from one image and every odd line from the other. The resulting retransformed image shows the characteristic artifact that was observed in the measurements (cf. Fig. 1A, 2B).

To confirm that the artifact arises from the difference in TR, four sequence variants were simulated in the following that included: 1) adaptation of the flip angle for every other excitation, 2) application of a correction factor within the k-space data before reconstruction, 3) reordering of the k-space data, and 4) a combination of reordering and correction factor. All simulations were performed with the following parameters corresponding to the values of the later performed static phantom measurements: TR_1_ = 4 ms, TR_2_ = 7 ms; T_1_ = 1420 ms; T*_2_ = 450 ms; TE = 3 ms.

#### 1) Flip angle adaptation

In [26], the application of two different flip angles to avoid the artifact was examined. Similar to the formula given in [24], a general formula for imaging with two TRs, TR_1/2_, and flip angles, θ_1/2_, can be derived (s. S1 Appendix):

$$S_{1}=M_{0}\;\cdot \frac{1+e^{-\frac{T_{R,2}}{T_{1}}}\cdot \left(\left(1-e^{-\frac{T_{R,1}}{T_{1}}}\right)\cdot \text{cos}\;\theta_{2}-1\right)}{1-\text{cos}\;\theta_{1}\cdot \text{cos}\;\theta_{2}\cdot e^{-\frac{T_{R,1}+T_{R,2}}{T_{1}}}}\;\cdot \;e^{-\frac{T_{E}}{T_{2}^{*}}}\;\cdot \;\text{sin}\;\theta_{1}$$ (Eq. 3)

and accordingly:

$$S_{2}=M_{0}\;\cdot \frac{1+e^{-\frac{T_{R,1}}{T_{1}}}\cdot \left(\left(1-e^{-\frac{T_{R,2}}{T_{1}}}\right)\cdot \text{cos}\;\theta_{1}-1\right)}{1-\text{cos}\;\theta_{1}\cdot \text{cos}\;\theta_{2}\cdot e^{-\frac{T_{R,1}+T_{R,2}}{T_{1}}}}\;\cdot \;e^{-\frac{T_{E}}{T_{2}^{*}}}\;\cdot \;\text{sin}\;\theta_{2}$$ (Eq. 4)

The condition S1 = S2 has to be met to preclude the artifact and can be solved numerically. By keeping θ_2_ fixed and varying θ_1_ from 1° to 90°, a flip angle θ_1_(θ_2_) that minimizes the signal difference can be determined. With the hereby determined flip angle θ_1_(θ_2_), the simulation was repeated by calculating two images by inserting the flip angle map in Equation (3) and (4), then combining the FT representations line-by-line before retransformation. This technique was only used for the simulations, not for the phantom measurements.

#### 2) k-space correction

Another way to influence the artifact is to apply a correction factor to the raw data in k-space. In order to avoid prolongation of total measurement time, only the center line of k-space can be acquired twice with both TRs to estimate the correction factor for all lines with lower signal. With a view to the later phantom measurements and in contrast to the flip angle adaptation, acquiring a correction factor would not depend on an a-priori estimation of the signal difference between TRs, which depends among others on T_1_ and the actually achieved flip angle distribution. To calculate the k-space correction factor, the mean ratio of the k-space center lines of the image with weak / strong signal (Eq. (1) and (2)) was determined. Then, every k-space line that was generated by the weaker signal image was multiplied by the inverse of this ratio. From this data a new image was reconstructed. K-space correction was used both in the simulations and static phantom measurements.

#### 3) Reordering

A completely different approach to reducing the impact of the artifact is to change the order in which the lines in k-space are acquired. Image contrast is determined by the data in the center of k-space. Applying a low-pass filter by shifting the lines generated by the weaker signal to the higher k-space frequencies while keeping the lines with stronger signal in the center, the image signal should increase and the artifact should be suppressed. Therefore, the magnitude-reduced lines were distributed equally to the uppermost / lowermost ends of the FT of the target before retransformation. Reordering was used in all later simulations and measurements.

#### 4) Reordering and k-space correction factor

For additional image correction, the same reordering described in the above case is combined with the k-space correction factor by multiplying the lines originating from the weaker signal with the factor determined in case 2. The combination of reordering and k-space correction was used in the simulations and static phantom experiments.

### Sequence Modifications

After simulation of the sequence variants described above, the methods were integrated into the sequence source code. In addition to the modifications described in [12], the following changes were made to the TFL sequence to examine the aliasing artifact:

1. For the estimation of the correction factor to be applied to the lines acquired with lower signal, the center line of k-space is acquired twice using both effective TRs. For reconstruction, only the acquisition with higher signal is used.
2. The k-space ordering of this sequence usually moves from the highest to the lowest k-space phase encoding step (or vice versa), which means that the center is acquired in the middle of the acquisition. For the reordering described in case 3, the lines with weaker signal are distributed to the higher areas in k-space. As an example for a resolution of 64, the RF pulse with the weaker signal is assigned to phase encoding steps −32 to −17 and +16 to +31, whereas the excitation with higher signal is assigned to −16 to +15 (including zero). The acquisition order would then be −32, −**16**, −31, −**15**,…**−1**, **−**17, **0**, 16, **1**…, **15**, 31 (RF with higher signal in bold font), which means that the center of k-space is still acquired in the middle of the overall acquisition. As long as the total number of acquired lines is a multiple of 4, this reordering scheme is valid for this sequence, although the number of GRAPPA reference lines must also be divisible by 4 to maintain a valid reordering scheme if parallel acquisition is used. The number and acquisition style of GRAPPA reference lines is the same as in the original TIAMO reconstruction [15].

### Measurement Configuration

#### Static phantom measurements

Examinations were performed on a 7T whole-body system (Magnetom 7T, Siemens, Erlangen, Germany) with a 32-channel transmit/receive head coil (Nova Medical, Wilmington, MA). A spherical vendor-provided polydimethylsiloxane oil phantom with T_1_ = 1420 ms was used. One slice was acquired in the isocenter with effective TR_1_ = 4 ms and TR_2_ = 7 ms using a TE = 3.07 ms, a bandwidth of 488 Hz/pixel, an acquisition matrix of 320 x 320, a field of view (FOV) = 300 mm x 300 mm, and a nominal flip angle of 80°. Saturation sequence block was applied every second k-line to evoke two different TRs, but the saturation RF pulse itself was applied with a voltage of 0 V to assure that the artifact is only generated by the difference in effective TR. The measurements were performed without using TIAMO or GRAPPA.

#### Dynamic phantom measurements

Additional measurements were performed using the same 32-channel head coil and a custom-built cylindrical phantom containing a tissue-simulating liquid (37.5% water, 56.5% sugar, 6% salt, ε_r_ = 46.3, σ = 0.8/V/m [15]) water-sugar solution and two tubes, one empty and one filled with the same liquid with 1:1000 Gadovist contrast agent (Bayer Schering Pharma AG, Berlin, Germany). A flexible tube of approximately 0.6 mm diameter was placed directly next to the outside surface of the phantom. The water running at constant flow velocity of approximately 0.2 m/s through the tube was driven by a custom-built water pump: an immersion pump (“outside pump EXTRA”, COMET-Pumpen KG, Kriftel, Germany) combined with a power regulator (M171 PMW power control, Kemo Electronic, Langen, Germany) to change the constant flow velocity, both driven by a standard 12 V direct current power source of a desktop PC. The water pump was operated outside of the RF room passing the tubes through waveguides into the bore of the MR system. Imaging parameters corresponded to [12, 13] and were identical to the in-vivo measurements given below. TIAMO and GRAPPA were applied for these measurements to be as close as possible to the in-vivo protocol. With the 32-channel head coil, just one transmission mode can be excited; hence, applying TIAMO just leads to averaging of both acquisitions.

#### Volunteer measurements

Examinations were performed with a 16-channel transmit/receive coil based on [27]. The same configuration as in [12, 13] was used. Five stripline meander elements were placed dorsally on the patient table and eleven meander elements were placed on a rigid, semicircular former above the table. A RF shimming system with 8 channels similar to [28] was used to drive the coil with an 8-channel variable power combiner (VPC) interfaced to a 16-channel Butler matrix [29]. Eight of the 16 inputs of the 16-channel Butler matrix corresponding to the highest transmit signal in the corresponding transmit modes were connected to the VPC [27]. In this way the 8-channel transmit system could be used to drive the 16-channel coil. Image acquisition was performed with an AngioSURF table [30, 31] of 2 m length by positioning the volunteer feet-first supine. The AngioSURF table can be moved manually to the desired body position through the RF coil, which remained stationary at the isocenter connected to the original patient table. For compliance with the International Electrotechnical Commission (IEC) guidelines, SAR calculations (CST Microwave Studio, Darmstadt, Germany) were performed in human adult male and female body models of the Virtual Family and the Visible Human [32, 33]. Full-wave simulations were applied with exact dimensions and characteristics of the 16-channel RF coil, and maximum permitted input power levels for each station were calculated from the simulations of the corresponding body models. Based on these simulations, a standardized SAR file was integrated into the SAR monitoring system [28].

Almost the same volunteer imaging protocol as in [12, 13] was used: a nominal flip angle of 80° and a FOV of 375 mm by 281 mm to acquire 60 transversal slices with 2 mm thickness using a matrix of 384 by 288 pixels and thus an in-plane resolution of approximately 1 mm^2^. Parallel image acceleration with a GRAPPA factor of 4 utilizing 32 integrated auto-calibration lines was used. A TR of 705 ms per slice, a TE of 3.81 ms, and a bandwidth of 1090 Hz/pixel were chosen. Image slices were acquired sequentially and in ascending order using flow compensation. No inversion RF pulses for contrast preparation were used. RF pulses for venous saturation were applied every second TR.

TIAMO imaging [15, 16] used the CP^+^ and CP^2+^ modes. The sequence was gated by using an acoustic cardiac triggering device in such a way that one complete slice was acquired after each trigger signal. Due to TIAMO, each slice has to be acquired twice to combine both modes. The two modes are acquired consecutively to ensure that the sparse saturation RF pulse is applied with the same mode as the two subsequent excitation pulses. After a single trigger signal, one complete slice is acquired with the first mode and the same slice excited with the second mode follows after the next trigger event.

#### Measurement Sequence

Using the spherical phantom, the results from the simulations were verified with normal ordering, with the k-space correction factor, with reordering, and with reordering combined with the k-space correction factor. To approach the contrast expected in MRA with defined and reproducible conditions, normal ordering and reordering were compared using the flow phantom with constant flow velocity. Finally, to verify that the behavior would not change when measuring in-vivo, e.g. due to pulsatile flow, normal ordering and reordering were examined in a healthy volunteer.

## Results

In Fig. 2, the simulation results are shown including the flip angle map (Fig. 2A) and the artifact that results when two different TRs are applied (Fig. 2B). A comparison of the effect of the different variants on the image can be found in Fig. 2C to 2E, while 2f shows the calculated flip angle for a direct adaptation of the excited signal. In Fig. 2G, the artifact signal intensity is given relative to the corresponding signal inside the phantom. For the different techniques, Fig. 2H shows the signal inside the phantom relative to the image with artifact (2b). The phantom signal of the artifact image is also plotted relative to the two images that were calculated by using only the stronger (Eq. (1)) and weaker (Eq. (2)) signal respectively.

Comparison with Fig. 2G (blue line) shows that the artifact signal increases—relative to the signal inside the phantom—with higher flip angles because the signal difference is greater at higher flip angles. The observed artifact signal corresponds to signal from within the phantom and appears at a location shifted in the phase-encoding direction. The calculations revealed that the artifact intensity depends mainly on the ratio TR_1_ / TR_2_ and the flip angle, but only minimally on T_1_ as long as TR << T_1_ (results not shown). The higher the flip angle, the greater the signal difference (Eq. (1) and (2)) and the greater the artifact signal. Looking at the signal inside the phantom, one can see in Fig. 2H that the increase in the artifact signal is reflected in the signal decrease within the phantom of the image with artifact relative to an image with strong signal only (Fig. 2H, dotted blue line). Simultaneously, the signal within the phantom of the image with artifact gets higher with higher flip angles relative to the image with weaker signal only (Fig. 2H, dashed blue line).

Using Equations (3) and (4), a flip angle θ_1_ was calculated that depends on θ_2_ (Fig. 2F). Only for small flip angles (here, approximately < 25°) is the signal difference small enough that the same flip angles can be chosen for both excitations. An excitation flip angle adaptation did not lead to an artifact-free image for the simulated non-homogenous flip angle distribution (Fig. 2A) as only for a single flip angle can the artifact be nulled (here exemplarily shown for 70° in Fig. 2C, 2G: red line). Although the artifact level could be reduced to less than 10% over the flip angle range below the adapted flip angle of 70°, there is an interval (< 47°) where the artifact level is even higher than in the uncorrected image. With this technique, the overall artifact level would be reduced if the flip angle range contained in the image is not as broad as in this calculation, i.e. 48° to 90° instead of 1° to 90° when optimizing for 70°. This range would be sufficient when looking at e.g. 7-T head imaging. Over a wide flip angle range, the signal within the phantom is approximately 10% higher relative to the image with artifact with a slight increase towards higher flip angles; however, below 5° there is a relative signal decrease of less than 10% (Fig. 2H, red line).

Similar to the flip angle adaptation, the k-space correction factor technique is able to reduce the artifact signal relative to the signal inside the phantom, but below a certain flip angle that shows maximal reduction, the artifact level again increases and even higher relative values are reached below about 29° (Fig. 2G, green line). The general course of the relative artifact signal is nearly the same as for the flip angle adaptation technique. The signal inside the phantom increases over the entire flip angle range: 4% for small flip angles with a slight decrease to approximately 3% for higher flip angles (Fig. 2H, green line).

**Fig 3:**
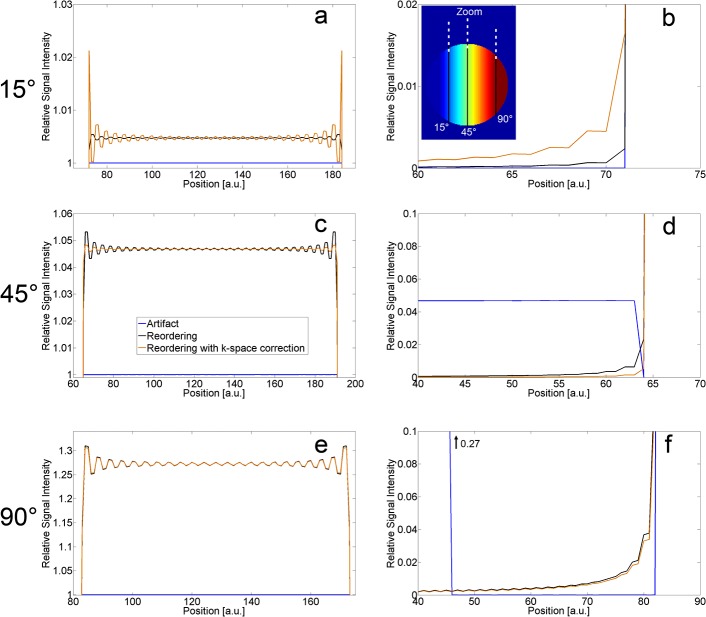
Ringing artifact. Ringing artifact generated by the reordering of k-space (black line) compared to the uncorrected artifact image (blue line) and with additional k-space correction factor applied (orange line). On the left (a, c, e) profiles through the images of Fig. 2B, E show the relative signal increase compared to the original artifact image within the phantom. On the right (b, d, f) the upper border of the phantom was zoomed in to show artifact signal outside. Note that the scaling of the axes is different.

Reordering of the k-space lines during acquisition prevents the aliasing artifact (Fig. 2E) almost completely (slight increase to max 0.2% at 90°), and the signal inside the phantom is increased (Fig. 2H, dashed black line). The signal of the reordered image approximates the signal increase that is observed comparing artifact image and image with strong signal only (Fig. 2H, dashed black line and dotted blue line). Direct comparison of the image formed by reordering and the image formed by the stronger signal only showed that the highest difference, observed at 90°, is still less than 5%. This means that reordering almost mimics the signal of an image with strong signal only. However, by reordering the k-space, a ringing artifact appears with increased amplitude towards higher flip angles (Fig. 3, black line). For 15°, the ringing amplitude is less than 0.1%, for 45° less than 0.6%, and for 90° less than 3%. For higher flip angles, ringing can also be observed outside the phantom (Fig. 3F).

If the k-space correction factor is applied to the weaker k-space lines after reordering, approximately identical relative artifact signal / signal within the phantom are reached as for the reordering technique alone; hence, these results not plotted in Fig. 2. The correction factor is able to reduce the amplitude of the ringing for medium flip angles (45°: Fig. 3C, D). For higher flip angles only a slight reduction is achieved (Fig. 3E, F), but for flip angles below 45°, the ringing amplitude is even increased by the k-space correction (15°: Fig. 3A, B).

For the phantom measurements, the flip angle adaptation was not further pursued, only the k-space correction factor was used to determine its impact on combining it with k-space reordering. A nominal flip angle of 80° was chosen to get high artifact signals, as the simulations showed that the artifact intensity increases with flip angle. As at 7 T the excitation profile is known to be inhomogeneous, a nominal flip angle of 80° was chosen to prevent flip angles higher than 90° that are normally not used for excitation with sequences based on gradient echo.

**Fig 4:**
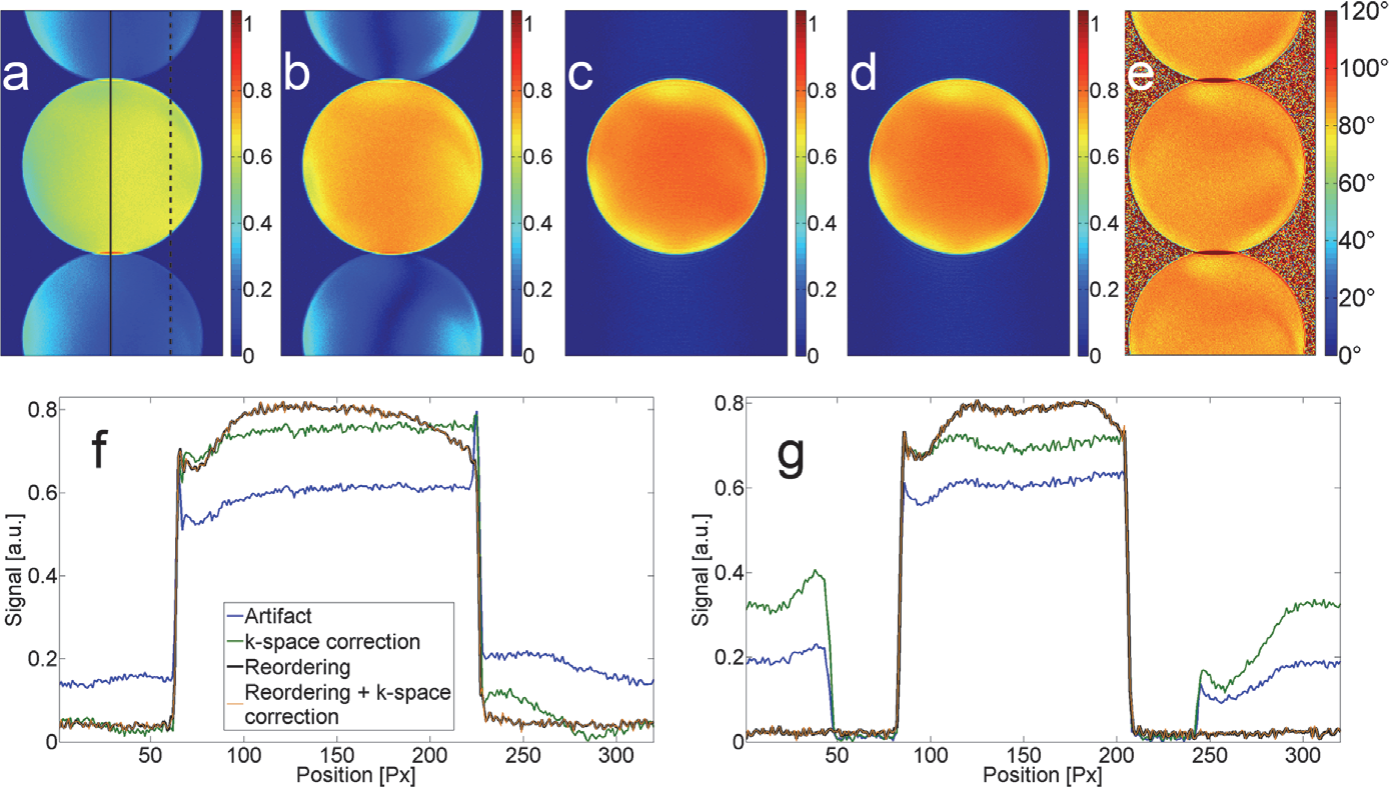
Static phantom measurements. Results of phantom measurements showing uncorrected artifact image (a), image with k-space correction (b), image with reordered acquisition (c), and image with reordering and k-space correction factor combined (d). (e) shows an estimation of flip angle obtained by reconstructing both signals individually and calculating a flip angle map as described for the AFI method [24]. In the lower row, profiles through the middle of the phantom (f) and on the right side of the phantom (g) are shown. Positions of the profiles are exemplarily shown in (a). Reordering and reordering combined with k-space correction are almost congruent (black and orange lines in f, g).

In Fig. 4, the results from the phantom measurements are shown. The images were not corrected for the receive sensitivity profile of the coil. In the upper row, the images with artifact (Fig. 4A) and with k-space correction factor (Fig. 4B) are shown. As in the simulations, the correction factor can only suppress the artifact partially (Fig. 4B). The reordering of k-space suppresses the entire artifact (Fig. 4C), while after combination with the k-space correction factor no relevant improvements were observable (Fig. 4D). Fig. 4E shows a flip angle estimation obtained by reconstructing both signals individually and calculating a flip angle map as described for the AFI method [24] from the two generated images. Both individual images show strong aliasing as only half the lines could be used for reconstruction. In the profiles through the middle of the phantom, the curves for reordering and reordering combined with k-space correction are almost identical (Fig. 4F, black and orange lines), but applying the correction factor increases the noise in the image by a factor up to approximately 2. The mean artifact signal (relative to the signal inside the phantom) was approximately 28% for the artifact image and 6% after reordering. In the profile on the right side of the phantom (Fig. 4G), one can clearly see that the k-space correction factor can also lead to an amplification of the artifact (Fig. 4G, green line). Both the correction factor and reordering were able to increase the signal within the phantom.

**Fig 5:**
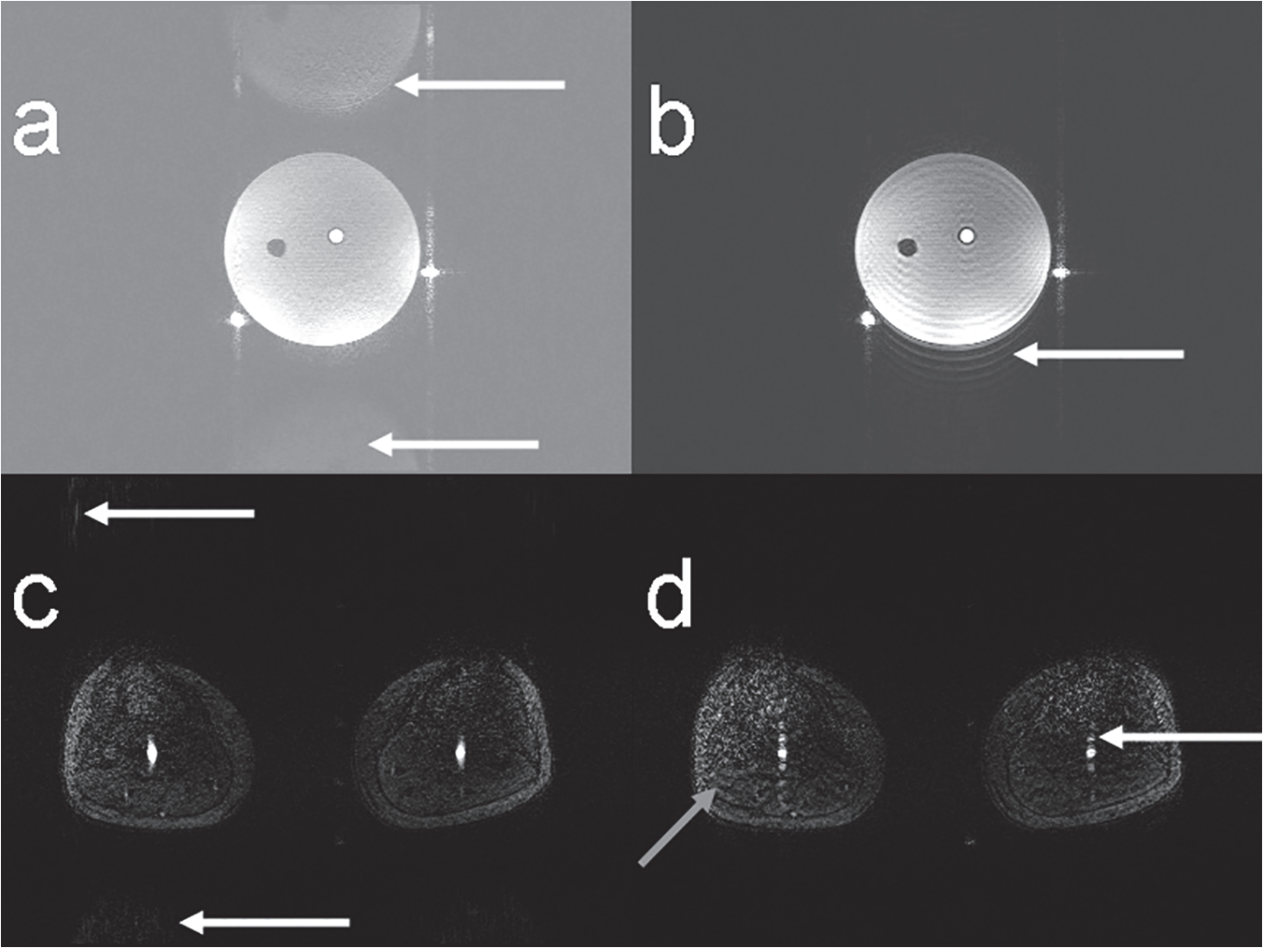
Flow phantom and volunteer measurements. Upper row: measurement results in the flow phantom. Only the tubes outside the phantom contained flowing water at constant velocity. The white arrows in (a) point to the aliasing artifact while in (b) the ringing artifact (arrow) is visible that originates from the reordering scheme. In the lower row, in-vivo images are shown with the aliasing artifact almost not visible ((c), arrows). Using a reordered acquisition led to slight ringing artifacts ((d), oblique grey arrow) and to multiple depiction of the arteries (white arrow). Image (c) and (d) are windowed identically.

In the upper row of Fig. 5, results from the measurements with the flow phantom are shown. Water was flowing with constant velocity only within the tubes outside the phantom. Using GRAPPA, the aliasing artifact was less pronounced (Fig. 5A). Applying reordering, the aliasing transforms into ringing within and outside the phantom, just as in the simulations (Fig. 3). The tubes inside the phantom also show slight ringing, while the tubes containing flowing water seem not to be affected by the reordered acquisition.

In the volunteer measurement (Fig. 5C), aliasing was less pronounced than in the aforementioned images, but still visible if windowing was chosen accordingly. No aliasing was observable in the reordered case, while ringing was slightly visible within the legs (oblique grey arrow in Fig. 5D) and slightly visible outside. The arteries, however, are depicted multiple times in the phase-encode direction while being only slightly dilated using standard ordering as reported in [12].

## Discussion

In contrast to the original TIAMO publication [15], the modes here were acquired consecutively rather than interleaved. Nevertheless, the same reconstruction as in the original publication could be used as exactly the same type of k-space data is acquired. Movement between the two separate TIAMO acquisitions is a potential problem; however, no such artifacts had been observed in previous volunteer / patient studies with the consecutive acquisition strategy. Also, T_1_ contrast may change if TIAMO is not acquired in an interleaved fashion, but this was not considered crucial for this angiographic application, where contrast between suppressed background tissue and hyper-intense vessels is the aim.

To reduce complexity and to concentrate on the hypothesized artifact origin, cardiac triggering and e.g. blood flow velocity changes were not considered for the simulations (or in the phantom measurements to verify the simulations). The purpose of this study was to demonstrate the definite origin of the artifact. Hence, cardiac triggering was excluded to show that it is not a prerequisite to generate this kind of artifact.

Of the compared simulated sequence variants, reordering of k-space during data acquisition was the only technique able to reduce the artifact intensity significantly over the entire flip angle range and to restore the signal within the phantom. Both flip angle adaptation and k-space correction nearly nulled the artifact signal only for a certain narrow range of flip angles. Although over this flip angle range the artifact signal is reduced, there are flip angles for which the artifact signal is even increased. For the flip angle adaptation, equal signals would also be achievable by increasing the flip angle θ_2_ instead of decreasing θ_1_ as investigated here. However, this approach would imply increasing SAR.

For the k-space correction, the flip angle which showed minimal artifact signal was approximately the mean flip angle over the entire phantom. The flip angle adaptation searches for flip angles for which both signals are equal. In contrast, the k-space correction factor tries to increase the level of the weaker signal to the level of the stronger signal (Eq. (2) and (1)). For a perfectly homogeneous excitation, both techniques would decrease the artifact signal to zero, but the signal inside the phantom would be higher with the k-space correction, since it would reach the signal of an image with strong signal only. Comparing FT representation of images with stronger and weaker signal for an inhomogeneous phantom (results not shown), one can see that the correction factor is not constant for every line as for a homogeneous phantom, but oscillates from line to line and also within every line. In fact, the artifact is distributed in both directions of k-space, and a correction factor defined only line-by-line would only correct in one direction. Only a point-wise correction could lead to reasonable results, which would require acquiring the image twice. When using a GRE sequence which reaches a steady state after a certain number of acquisitions, the correction factor would deviate additionally for the lines which are acquired before reaching the steady state.

The signal of the reordered image approximates the signal of the image with strong signal only. This behaves as expected because the inner k-space is filled by the stronger signal acquisitions due to the reordering. The oscillation that can be seen in Fig. 2H (dashed black line) is due to the ringing artifact that can be observed at the borders and throughout the entire phantom for higher flip angles (Fig. 3). Suppressing the aliasing artifact by reordering the k-space in this way leads to a ringing artifact which is caused by the abrupt change in signal intensity at a certain threshold in k-space due to the lines with weaker signal. Inverting the reordering by placing the weaker signals in the center of the phantom led to the same ringing artifact besides reducing the signal within the phantom (results not shown). The aliasing artifact observed here and in [11, 12] is weaker than the known case of acquiring only every second line. Similarly, the ringing artifact caused by reordering is also weaker than it would be if caused by Gibbs ringing where only the inner k-space is acquired and zero-filling is used for the outer parts of k-space [34, 35]. For the simulations performed here, if the higher frequencies are filled with zeroes instead of with the weaker signal, Gibbs ringing is visible for all flip angles and oscillations of maximally 7% of the mean signal within the phantom are observable; with reordering of the weaker signals instead, only < 0.5% for 15° and 3% for 90° are reached.

Combining reordering with the k-space correction factor led to almost identical results regarding relative artifact signal and signal intensity within the phantom (hence not shown in Fig. 2). The same was observed for the ringing artifact for higher flip angles (Fig. 3E, F), while for lower flip angles the amplitudes of the ringing were even increased (Fig. 3A, B). Only for medium flip angles (e.g. 45°, Fig. 3C, D) was ringing clearly reduced. The reason is that 45° is quite near to the flip angle of approximately 40° in Fig. 2G that shows least artifact signal for the k-space correction factor. As is observed for the aliasing artifact with k-space correction factor in an inhomogeneous excitation, combining reordering with a k-space correction factor is not useful as the ringing artifact is reduced only in some parts of the flip angle range while it is increased in other parts. Another way to correct k-space for the ringing would be to apply some kind of filtering, e.g. a Tukey-window which can be used to reduce Gibbs ringing [34].

Although ringing is observed with reordering, reordering does not depend on the choice of one particular flip angle for which the artifact disappears, which is in strong contrast to the k-space correction factor or flip angle adaptation.

The phantom measurements of k-space correction, reordering, and reordering combined with k-space correction provided the same results that were achieved in the simulations. k-space correction seems to provide a flatter profile within the phantom, but this is most probably due to signal energy that is projected outside of the phantom, generating the aliasing artifact (Fig. 4F, green line).

As the sequence that was applied in [12, 13] uses two TRs, it actually acquires half the lines that are acquired when applying an actual flip-angle imaging (AFI) sequence [24]. The signal difference that produces the artifact can also be used to estimate the flip angle if the lines with stronger signal and the lines with weaker signal are reconstructed individually. With the formula given in [24], a flip angle map can be calculated using these two images (Fig. 4E). The result shows strong aliasing because the two individual images are reconstructed from two sets of data undersampled by a factor of two.

Although the aliasing artifact is weaker in-vivo than in the phantom measurements (Fig. 5A, C), the ringing predicted by the simulation could still be observed (Fig. 5D). The arteries, however, showed an artifact that cannot be explained by mere ringing as it was not visible with constant flow (Fig. 5B). Due to the multiple ghosting of the arteries in the images with reordering (Fig. 5D), this variant should not be used for suppressing the aliasing artifact. As this artifact was not observed when measuring with constant flow (Fig. 5B), the reason for the artifact must lie with the pulsatile flow in vivo. With the reordering technique, the inner lines of k-space are not acquired consecutively but interleaved with lines from the outside, extending the acquisition of the inner k-space over a longer period of time. This makes the acquisition with reordering more prone to changes in blood flow velocity.

Blood flows quite fast at the beginning of the cardiac cycle and then reduces its velocity to be quite constant before the next heart beats accelerates it again. This effect is visible as the slight vessel dilation in the image with aliasing artifact (Fig. 5C). Also, a very similar artifact was observed in some images for the FLASH sequence in [11]. In that work, image acquisition with the FLASH sequence was performed without cardiac triggering, which means that depending on the exact time the center of k-space is acquired either relatively constant blood flow or a relatively strong change in blood flow velocity is experienced, the latter leading to the slight vessel dilation.

The purpose of this study was to examine the origin of the artifact that occurred with the modified TFL sequence and the parameters that were used in previous volunteer and patient studies [11–13]. Hence, in this publication different methods were implemented to test the hypothesized origin and, as a byproduct, to observe their (dis-)advantages for imaging. The methods discussed in this work are not suited for sufficient suppression of the aliasing artifact. One possibility to suppress the artifact would be to apply a partial Fourier factor and use the thereby gained measurement time reduction to apply the saturation RF pulse in every line. However, due to the fact that coronal MIPs are used when looking at the images [13], the aliasing artifact in the phase-encode direction is not disturbing. With the current system hardware, only 8 kW of RF amplifier peak power are available, of which only approximately half is available at the coil. In a previous study, the flip angle achievable with the saturation pulses used in the current study was found to be only roughly 20° [13]. With higher amplifier power or by prolonging the duration of the saturation pulses to increase their flip angle, a more sparse application of the saturation pulses would presumably still lead to sufficient venous suppression due to the higher achieved flip angles and thus would be beneficial in reducing the artifact. Whether the use of a higher partial Fourier factor or amplifier power would preserve the angiography contrast and general image quality would have to be evaluated in further volunteer measurements together with a radiologic evaluation; this would be a study with a different focus that we may pursue in the future.

In conclusion, the simulation and phantom measurements confirmed that the origin of the observed aliasing artifact lies—as hypothesized—in the periodic signal variation due to the two effective TRs experienced by the spins during acquisition.

## Supporting Information

S1 Appendix Formula for flip angle correction. (DOCX)
