# Supplementary material for: Investigation of the Saturation Pulse Artifact in Non-Enhanced MR Angiography of the Lower Extremity Arteries at 7 Tesla
Source: PLoS One. 2015 Mar 18;10(3):e0119845. doi: 10.1371/journal.pone.0119845 (PMC4364710; doi:10.1371/journal.pone.0119845)
Supplement: S1 Appendix — (DOCX) [file pone.0119845.s001.docx]

**S1 Appendix. Formula for flip angle correction.**

In the steady-state, for $0^{\circ}\leq\theta_{1},\theta_{2}\leq90^{\circ}$ the magnetization has to satisfy the following relations (Fig. 1b):

1. $M_{2}^{+}=M_{2}^{-}\cdot\cos\theta_{2}$
2. $M_{2}^{-}=M_{1}^{+}\cdot e^{-\frac{T_{R,1}}{T_{1}}}+M_{0}\left( 1-e^{-\frac{T_{R,1}}{T_{1}}} \right)$
3. $M_{1}^{+}=M_{1}^{-}\cdot\cos\theta_{1}$
4. $M_{1}^{-}=M_{2}^{+}\cdot e^{-\frac{T_{R,2}}{T_{1}}}+M_{0}\left( 1-e^{-\frac{T_{R,2}}{T_{1}}} \right)$

Inserting (2) into (1) gives:

$$M_{2}^{+}=M_{1}^{+}\cdot e^{-\frac{T_{R,1}}{T_{1}}}\cdot\cos\theta_{2}+M_{0}\left( 1-e^{-\frac{T_{R,1}}{T_{1}}} \right)\cdot\cos\theta_{2}$$

Inserting this into (4) gives:

$$M_{1}^{-}=M_{1}^{+}\cdot e^{-\frac{T_{R,1}}{T_{1}}}\cdot e^{-\frac{T_{R,2}}{T_{1}}}\cdot\cos\theta_{2}+M_{0}\left( 1-e^{-\frac{T_{R,1}}{T_{1}}} \right)\cdot e^{-\frac{T_{R,2}}{T_{1}}}\cdot\cos\theta_{2}+M_{0}\left( 1-e^{-\frac{T_{R,2}}{T_{1}}} \right)$$

Inserting (3) into this result gives:

$$M_{1}^{-}=M_{1}^{-}\cdot e^{-\frac{T_{R,1}}{T_{1}}}\cdot e^{-\frac{T_{R,2}}{T_{1}}}\cdot\cos\theta_{1}\cdot\cos\theta_{2}+M_{0}\left( 1-e^{-\frac{T_{R,1}}{T_{1}}} \right)\cdot e^{-\frac{T_{R,2}}{T_{1}}}\cdot\cos\theta_{2}+M_{0}\left( 1-e^{-\frac{T_{R,2}}{T_{1}}} \right)$$

Finally, solving the equation for$M_{1}^{-}$ yields:

$$M_{1}^{-}=M_{0}\cdot\frac{1+e^{-\frac{T_{R,2}}{T_{1}}}\cdot\left( \left( 1-e^{-\frac{T_{R,1}}{T_{1}}} \right)\cdot\cos\theta_{2}-1 \right)}{1-\cos\theta_{1}\cdot\cos\theta_{2}\cdot e^{-\frac{T_{R,1}+T_{R,2}}{T_{1}}}}$$

And accordingly:

$$M_{2}^{-}=M_{0}\cdot\frac{1+e^{-\frac{T_{R,1}}{T_{1}}}\cdot\left( \left( 1-e^{-\frac{T_{R,2}}{T_{1}}} \right)\cdot\cos\theta_{1}-1 \right)}{1-\cos\theta_{1}\cdot\cos\theta_{2}\cdot e^{-\frac{T_{R,1}+T_{R,2}}{T_{1}}}}$$

To ensure correct image acquisition without artifact, the signals from both excitations with different $T_{R}$ have to be the same. Since the tissue parameters are the same in both cases, the flip angles have to satisfy:

$$M_{2}^{-}\cdot\sin\theta_{2}=M_{1}^{-}\cdot\sin\theta_{1}$$

$$\left( 1+e^{-\frac{T_{R,1}}{T_{1}}}\cdot\left( \left( 1-e^{-\frac{T_{R,2}}{T_{1}}} \right)\cdot\cos\theta_{1}-1 \right) \right)\cdot\sin\theta_{2}=$$

$$\left( 1+e^{-\frac{T_{R,2}}{T_{1}}}\cdot\left( \left( 1-e^{-\frac{T_{R,1}}{T_{1}}} \right)\cdot\cos\theta_{2}-1 \right) \right)\cdot\sin\theta_{1}$$

This can be solved numerically. Assuming realistic values of $T_{R,1},T_{R,2}\ll T_{1}$ and ${T_{R,2}\leq T}_{R,1}\leq3T_{R,2}$, $\theta_{2}$ can be approximated for a given $\theta_{1}$ (in radians) and $m=\frac{T_{R,2}}{T_{1}}$ by using this equation:

$$\theta_{2}=0.7979\cdot\theta_{1}\cdot e^{0.4651m+0.1771\theta_{1}}-0.3225m\cdot{\theta_{1}}^{2}-0.2282m^{2}\cdot\theta_{1}$$
